# Bronchiectasis in Children: Current Concepts in Immunology and Microbiology

**DOI:** 10.3389/fped.2017.00123

**Published:** 2017-05-29

**Authors:** Susan J. Pizzutto, Kim M. Hare, John W. Upham

**Affiliations:** ^1^Child Health Division, Menzies School of Health Research, Darwin, NT, Australia; ^2^Department of Respiratory Medicine, Princess Alexandra Hospital, Brisbane, QLD, Australia; ^3^School of Medicine, The University of Queensland, Brisbane, QLD, Australia

**Keywords:** bronchiectasis, children, immunology, microbiology, chronic suppurative lung disease

## Abstract

Bronchiectasis is a complex chronic respiratory condition traditionally characterized by chronic infection, airway inflammation, and progressive decline in lung function. Early diagnosis and intensive treatment protocols can stabilize or even improve the clinical prognosis of children with bronchiectasis. However, understanding the host immunologic mechanisms that contribute to recurrent infection and prolonged inflammation has been identified as an important area of research that would contribute substantially to effective prevention strategies for children at risk of bronchiectasis. This review will focus on the current understanding of the role of the host immune response and important pathogens in the pathogenesis of bronchiectasis (not associated with cystic fibrosis) in children.

## Introduction

Bronchiectasis is a complex chronic respiratory condition traditionally characterized by recurrent infection, airway inflammation, and progressive decline in lung function. The defining symptom common to children with bronchiectasis is chronic wet cough. Region-specific studies suggest that geographic locality and socioeconomic environment play a large role in determining the likely etiology of bronchiectasis in children. Cystic fibrosis, primary immune deficiency, defects in mucociliary clearance mechanisms (primary ciliary dyskinesia, congenital malformations), and aspiration of a foreign body increase susceptibility to respiratory infection and are known to be associated with bronchiectasis in children. However, on a global scale, lower respiratory infection in the absence of known underlying conditions accounts for the greatest number of bronchiectasis cases (1). The focus of this review will be bronchiectasis with no known underlying disorder in children.

In underprivileged populations, including populations within affluent societies (such as Indigenous populations of Australia, New Zealand, and Alaska), severe lower respiratory infection early in life is the most likely cause of bronchiectasis (2–4). Globally, it is estimated that severe bacterial or viral pneumonia accounts for approximately 60% of the cases of postinfection pediatric bronchiectasis. Measles and tuberculosis combined account for 25% (1). Although pneumonia is a significant risk factor for bronchiectasis, only a proportion of children develop bronchiectasis following an episode of pneumonia (5). The mechanisms involved in progressing from acute lower respiratory infection to chronic inflammation and persistent infection are poorly understood.

Early diagnosis and intensive treatment protocols can stabilize or even improve the clinical prognosis of children with bronchiectasis (6, 7). Over the decades, considerable advances have been made in identifying the pathogens associated with recurrent or chronic infection. However, understanding the host immunologic mechanisms that contribute to recurrent infection and prolonged inflammation has been identified as an important area of research that would contribute substantially to effective prevention strategies for children at risk of bronchiectasis (8, 9).

This review will begin by exploring the role of the child’s immune response in establishing an environment conducive to the recurrent infection and chronic inflammation characteristic of bronchiectasis. This will be followed by a discussion of the important pathogens associated with bronchiectasis in children and the obstacles in treating and preventing these infections. The review will conclude with “the road forward” areas of research identified by the authors as important for the advancement of understanding and addressing the pathogenesis of bronchiectasis in children.

## Pathophysiology of Bronchiectasis

Over recent years, considerable efforts have been made to understand airway inflammation associated with bronchiectasis. Some parallels exist between bronchiectasis and other chronic respiratory disorders, including cystic fibrosis and chronic obstructive pulmonary disease (COPD), in relation to airway inflammation. As a result, inflammatory processes involved in cystic fibrosis and COPD are sometimes used in an attempt to understand the pathophysiology of bronchiectasis. However, cystic fibrosis and COPD have distinctly different etiologies from bronchiectasis in children and extrapolating data, particularly to the pediatric setting, should be done with caution. Nevertheless, similarities between the chronic respiratory conditions suggest that bronchiectasis arises from exaggerated and/or dysregulated inflammation in response to challenge from respiratory pathogens. Thus, the “vicious circle” hypothesis of self-perpetuating infection, inflammation, and tissue damage first described by Cole in 1986 (10) remains the most likely explanation for the pathogenesis of bronchiectasis. What remains in question is, what causes the highly controlled immune response to become dysregulated?

The innate inflammatory response is one of the rapid response units of the immune system. When pathogens penetrate the physiological barriers, such as the epithelium, a series of highly regulated cellular and non-cellular events coordinate to rapidly contain the infection. In addition to its role as a first response unit, the inflammatory response also orchestrates the initiation of the pathogen-specific adaptive response. When functioning optimally, the initial inflammatory process resolves as rapidly as it begins, maintaining tissue homeostasis. Dysfunction, whether an exaggerated initial response or delay in resolution, may cause an accumulation of potent cytotoxic compounds that can damage host tissue and provide an environment conducive to further infection.

## Airway Inflammation

Difficulties associated with obtaining lower airway specimens from young children have precluded comprehensive study of the localized inflammatory mechanisms contributing to bronchiectasis. As a result, much of the current knowledge of airway inflammation in children with bronchiectasis is derived from small, cross-sectional studies, retrospective chart reviews and extrapolation of data from studies of bronchiectasis in adults. Despite these limitations, studies of bronchiectasis in children from various environments consistently show neutrophilic inflammation of the airways (11–13) as well as elevated levels of associated proinflammatory cytokines (IL-8, TNFα, IL-1β, and IL-6) (11, 14–16) and anti-microbial compounds (IP-10 and LL-37) (16), consistent with an inflammatory response to bacterial assault. However, in addition to neutrophilic inflammation, eosinophilic inflammation has been described in young Australian Indigenous children newly diagnosed with bronchiectasis (13), suggesting complex inflammatory pathways may contribute to bronchiectasis.

Neutrophilic airway inflammation is also characteristic of bronchiectasis in adults. Neutrophil counts and inflammatory cytokines including IL-1β, IL-6, IL-8, and TNFα are elevated during periods of infective exacerbation (17, 18) and these key markers of inflammation continue to persist during periods of apparent clinical stability (17, 19). Increased levels of inflammatory markers in the lungs are associated with more severe respiratory symptoms, including poorer lung function (11, 20–22) and correlate positively with the anatomical extent of bronchiectasis (20).

Pediatric studies are important to our understanding of the pathogenesis of bronchiectasis. Inflammation in the absence of bacterial infection in children with relatively newly recognized disease could mean that neutrophilic inflammation is an indication of abnormal immune regulation, rather than a symptom of chronic infection.

## Systemic Inflammation

Although traditionally recognized as an inflammatory disease of the airways, there is a growing body of evidence that bronchiectasis may also include a systemic inflammatory component. While studies in children have found limited indication of systemic inflammation using standard clinical markers of inflammation [C-reactive protein (CRP), total white cell count, protein, platelets] (16), a study of 22 children found that children with bronchiectasis (clinically stable) had a higher proportion of circulating proinflammatory lymphocytes (producing TNFα, IFN-γ, perforin, and granzyme), compared with children without suppurative lung conditions (23). This study was small and cross-sectional in design; however, it raises the possibility that a low-grade level of systemic inflammation is present in children with bronchiectasis and not currently detected by standard investigations.

Systemic inflammation is relatively common in adults with bronchiectasis. Elevated levels of circulating inflammatory cells (neutrophils and total white cell count) as well as soluble serum mediators [including transforming growth factor (TGF)-β, CRP, fibrinogen, and soluble adhesion molecules] have been described in adults with long-standing disease (17, 24–27). A high level of systemic inflammation is associated with an accelerated decline in lung function (28). Therefore, novel markers to monitor low-grade systemic inflammation may be important for managing and preventing progression of disease in children.

## The Role of Neutrophils in Bronchiectasis

Neutrophilic inflammation of the airways is often present in bronchiectasis, both in the presence and apparent absence of bacterial infection. In the healthy lung, the neutrophil is one of the key cells involved in driving the inflammatory response against invading pathogens. Neutrophils are recruited from peripheral circulation by local populations of macrophages and neutrophils. Circulating neutrophils rapidly migrate to the airways in response to proinflammatory mediators including IL-1β, IL-8, and TNFα. The primary role of the neutrophil is to eliminate the invading pathogen, first by phagocytosis, followed by release of an arsenal of antimicrobial products within the phagosome. When significantly stimulated, neutrophils degranulate and deploy potent proteases (including neutrophil elastase, cathepsin, and myeloperoxidase). Inadvertently, the release of these proteases can also degrade matrix proteins of the host’s airway walls and promote further inflammation. In the healthy lung, this process is tightly regulated to prevent excessive damage to host tissue. Impaired function and/or dysregulation of neutrophils are proposed as important in the pathogenesis of bronchiectasis.

There are currently no data regarding neutrophil function in children. However, impaired neutrophil function has been described in adults with bronchiectasis, and this is associated with more severe disease (29–31). King and colleagues (30) demonstrated a high prevalence of impaired bacterial-specific oxidative burst function by circulating neutrophils in a large group of adults with bronchiectasis. Furthermore, patients with a low capacity for neutrophil-generated oxidative burst also demonstrated a reduced capacity for intracellular bactericidal activity by the neutrophils. These data, however, were not reflected in two small studies of adult bronchiectasis where no impairment in oxidative burst by circulating neutrophils was found (29, 32), although impairment of airway neutrophils was observed (29). The discrepancies between these data may be attributed to the methods used to induce oxidative burst (bacterial phagocytosis versus synthetic peptide) or the clinical characteristics of the study cohorts (age and severity of disease). In the study by King and colleagues, phagocytosis and intracellular killing were investigated using a bacterial challenge, which may represent an alternate and more accurate pathway of activation compared with using peptide as the challenge. Despite the conflicting data, both studies found evidence of impaired microbicidal activity, albeit in different populations of neutrophils, suggesting that functional phagocytosis accompanied by impaired intracellular killing may be one strategy employed by pathogens to establish an intracellular niche and avoid host clearance mechanisms. The possibility that impaired phagocytosis and intracellular killing mechanisms correlate with disease severity highlights the need to investigate the role of neutrophil function in the progression of bronchiectasis in children who are in the early stage of chronic disease.

### Eosinophils

Eosinophils comprise a small but potent proportion of the leukocyte population in circulation and in the lungs. Recruitment and activation of eosinophils are associated with several respiratory disorders including asthma and eosinophilic bronchitis, as well as parasite infection. Although rarely reported in association with bronchiectasis, our studies have identified a high prevalence of airway eosinophilia in Australian Indigenous children with bronchiectasis, which correlates with circulating eosinophils (13, 16). Limited investigations implicated a possible role for viruses in elevated airway eosinophils (16); however, it is likely that airway eosinophilia in children with bronchiectasis has multiple etiologies, including parasite infection, coexistent asthma, and hypersensitivity to fungi. In adults with chronic airway diseases, including bronchiectasis, eosinophilia is thought to be associated with more severe disease (33). Therefore, it is important to understand the etiology of airway eosinophilia and its contribution to the perpetuation of chronic inflammation and the pathogenesis of bronchiectasis.

### Macrophages

Macrophages are phagocytic cells with multiple phenotypes. They are the most abundant cell in the uninflamed lung, typically comprising 85–95% of the cellular profile of bronchoalveolar lavage (BAL) fluid (34). They reside in and around interstitial tissue (interstitial macrophages) and within the surfactant fluid lining the alveoli (alveolar macrophages).

Hodge and colleagues recently showed, for the first time, alveolar macrophage dysfunction in children with bronchiectasis (15). Compared with control children, alveolar macrophages from children with bronchiectasis had a reduced capacity for both efferocytosis of bronchial epithelial cells and phagocytosis of non-typeable *Haemophilus influenzae* (NTHi). Impaired efferocytosis of apoptotic epithelial cells and neutrophils has also been described in adults with asthma and COPD (35, 36). Efficient efferocytosis is paramount in preventing secondary necrosis and the release of toxic factors into the lung microenvironment. An impaired ability to clear both apoptotic host cells and pathogens would likely contribute to an environment conducive to tissue damage and persistent infection and the pathogenesis of bronchiectasis.

## Adaptive Immune Responses

Bacterial infection plays an important role in the pathogenesis of bronchiectasis in children. Severe lower respiratory infection during infancy is a significant risk factor for bronchiectasis (37), and idiopathic bronchiectasis in adults is often traced back to a history of lower respiratory infection in childhood (38). NTHi is the pathogen most commonly associated with bronchiectasis [as reviewed in Grimwood 2011 (9)]. However, NTHi is also present as a commensal organism in children without respiratory disease (39, 40). Despite the dual existence of NTHi as commensal organism and important respiratory pathogen (41), little is known about the development of natural immunity to NTHi. While it remains undisputed that the localized physiologic characteristics of bronchiectasis itself contribute to an environment supportive of recurrent and persistent infection (dilated airways, excessive mucus retention, dysfunctional cilia), complementary studies in children (42) and adults (43–45) have shown that the adaptive immune response, particularly the cell-mediated immune response, is an important factor contributing to recurrent respiratory infection with NTHi.

## Cell-Mediated Immune Responses

*In vitro* challenge assays of blood mononuclear cells indicate that the cell-mediated immune response to NTHi may be compromised in children with bronchiectasis. This was demonstrated by a reduced capacity to produce IFN-γ in response to NTHi (42). Importantly, this compromised cell-mediated immune response was strongly associated with airway inflammation, specifically, elevated levels of IL-1β and IL-6 (16), indicating a possible link between localized inflammation and systemic adaptive immunity. The mechanisms driving the association between airway inflammation and NTHi-driven IFN-γ in children with bronchiectasis have not been determined. However, IL-1β and IL-6 are integral to the initiation and phenotype of the adaptive immune response. IL-1β drives the inflammatory cascade by localizing neutrophils and promoting the production of inflammatory modulators such as IL-6 and IFN-γ-inducible protein 10 (IP-10; CXCL10). IL-6 plays a complex role in the inflammatory response, from promoting inflammation to wound healing. Dysregulation of IL-6 pathways is associated with chronic inflammation (46). In addition to its inflammatory modulating properties, IL-6 is integral to initiating the adaptive response and in directing its primary phenotype. In the lung, IL-6 polarizes the adaptive immune response in favor of the humoral response by inhibiting IL-12 production (47, 48) and by promoting the differentiation of B-cells into antibody-producing plasma cells (49). These data suggest that IL-6 and IL-1β may be more than simply markers of inflammation, but rather indicative of suboptimal T-helper pathways that result in impaired clearance mechanisms and persistent infection. Collectively these data support an association between impaired cell-mediated immune responses to NTHi, dysregulated airway inflammation, and the pathogenesis of bronchiectasis in children.

These data in children complement extensive studies of NTHi-specific immune responses in adults (43, 44) to show that a Th1 polarized cell-mediated immune response contributes to protective immunity against NTHi. In these studies, King and colleagues showed that circulating CD4+ T-helper cells from healthy adults responded to an *in vitro* NTHi challenge with increased expression of IL-2 and IFN-γ, in a predominantly classic Th1 manner. In contrast, the cytokine response from adults with bronchiectasis and chronic NTHi infection was polarized in favor of IL-4 and IL-10 and complemented by low expression of IL-2 and IFN-γ.

Several factors may contribute to the differences in cytokine profiles generated in response to NTHi, including a reduction in the size of the pool of CD4+ memory T-cells. However, King and colleagues (44) showed that polarization of cytokine profiles was in direct relation to a skewing of the cytokines produced, rather than an overall reduction in the absolute number of CD4+ T-cells. In addition to CD4+ T-cells, CD8+ cytotoxic T-cells have been implicated in chronic infection with NTHi (44). CD8+ T-cells have the capacity to switch between IFN-γ (Th1) and IL-4 (Th2) polarized responses (50). CD8+ phenotype switching has not been investigated with respect to chronic NTHi infection; however, CD8+ T-cells from adults with bronchiectasis demonstrated a non-specific capacity to produce IFN-γ that was not realized in response to a specific challenge with NTHi (44). Phenotype switching is one mechanism that may explain this.

Collectively, these studies suggest that children and adults with bronchiectasis likely have the necessary cell-mediated immune architecture to respond to NTHi. Children with recent onset disease and adults with established bronchiectasis have a universal capacity to produce IFN-γ that is similar to healthy controls, but fail to do so in response to NTHi. This raises the possibility of using therapeutic strategies, such as vaccination, to improve immunity to NTHi by promoting the protective Th1 response. Data in support of this strategy showed that in children with bronchiectasis, the capacity to produce IFN-γ in response to NTHi was highest in those who had received three or more doses of a pneumococcal conjugate vaccine that contained *H. influenzae* protein D (PHiD-CV) (51), a surface protein expressed by *H. influenzae*, not present in the HiB vaccine (52). Protein D has adjuvant properties that likely contributed to the higher cytokine responses in the PHiD-CV-vaccinated children.

## Humoral Immune Responses

The role of the humoral response in the pathogenesis of bronchiectasis is unclear. There are considerable published data showing chronic colonization and active infection of the lower airways with NTHi, despite the presence of an active humoral immune response (42, 51, 53–55). Adults with chronic respiratory conditions, including bronchiectasis, chronic bronchitis, and COPD, also exhibit a comprehensive humoral immune response against NTHi, with ample amounts of specific total IgG, IgG subclasses, IgA and IgM present in circulation (43, 45, 56). These antibodies are functional against NTHi *in vitro* and, when combined with serum complement, form a potent NTHi-clearance mechanism. Although the amount of circulating immunoglobulin is age dependent, children also appear to be proficient at producing NTHi-specific IgG (42, 51, 57, 58). It is thought that this strong, universal humoral response is one of the main reasons why NTHi rarely causes systemic infection. However, high systemic antibody levels do not appear to correlate with protection from respiratory infections (42, 45).

Secretory IgA is the main immunoglobulin associated with mucosal immunology. While data on NTHi-specific secretory IgA are lacking, a small study of 25 adult patients indicated that deficient secretory IgA was not associated with bronchiectasis or chronic bronchitis (56). In contrast, *in vitro* studies have shown that NTHi produces human IgA proteases that may facilitate its internalization and persistent survival within lung epithelial cells (59). It is plausible that impaired antibody function rather than deficient levels of antibody may contribute to recurrent infection with NTHi in bronchiectasis. Alternatively, the presence of bacterial biofilm or host bronchial secretions may impede the activity of antibodies; however, there are no data to confirm or refute this in children or adults with bronchiectasis.

Collectively, these data indicate that chronic infection with NTHi is unlikely attributed to an overall deficiency in antibody production. However, NTHi is a heterogeneous species and can induce the production of strain-specific antibodies (54, 60). Thus, it has been suggested that antibody specificity may explain the inability of a previous infection to protect against a future infection with NTHi. Contrary to this hypothesis, a considerable degree of functional antibody cross-reactivity has been demonstrated between different NTHi strains (45). Furthermore, in children and adults, there is a high turnover of strain carriage, and multiple strains are often carried concurrently (61, 62). Hence, it would be expected that a high level of antibody diversity is circulating at any one time, which should significantly restrict the number of infections regardless of strain specificity. These data do not preclude the role of humoral immunity in protection against respiratory infections with NTHi as humoral immunity likely plays a significant role in protection from systemic and airway disease. However, high prevalence of recurrent infection in the presence of high levels of antibody supports the argument that an increased susceptibility to infection with NTHi is more closely linked to the cell-mediated immune response than the humoral immune response.

## Persistent Infection and Bronchiectasis

Respiratory pathogens employ a variety of strategies to avoid clearance by host defense mechanisms. When successful, these strategies inhibit the host from effectively clearing infection and contribute to an environment supportive of chronic infection and associated inflammation. Some of the primary strategies employed by common respiratory pathogens include formation of protective structures such as biofilm (*H. influenzae, Pseudomonas aeruginosa, Staphylococcus aureus*, and *Streptococcus pneumoniae*) (63, 64), secretion of immune-blocking agents such as IgA proteases (*H. influenzae*) (65), and the secretion of toxins which damage mucus-clearing structures (including cilia) of the epithelium (66). Secreted proteases can damage the structure of the bronchial wall, including cilia, hampering sputum clearance from the lungs and promoting inflammatory processes by the host. Biofilm has been reported in the lower airways of children with bronchiectasis (67) and can impede the action of antibiotics (68). Some pathogens associated with chronic respiratory infections, including *H. influenzae* and *Mycobacterium tuberculosis*, can avoid the host humoral response by manipulating the host’s own phagocytic cells; hijacking antimicrobial mechanisms and establishing an intracellular niche (69).

## Bacterial Pathogens

Studies reporting bacteria associated with bronchiectasis in children are listed in Table 1. *H. influenzae* (specified as non-typeable, NTHi, in three studies) was the most common pathogen identified, followed by *S. pneumoniae* and *Moraxella catarrhalis* (2, 12, 70–75). These three species are commonly associated with acute exacerbations in adults with bronchiectasis (76, 77). While less common, combined results from all pediatric studies indicate that *P. aeruginosa* and *S. aureus* have similar prevalence to *M. catarrhalis*, although results are difficult to compare given the different specimen types used (Table 1). The prevalence of *H. influenzae, S. pneumoniae*, and *M. catarrhalis* in bronchiectasis forms the basis of empiric antibiotic therapy to treat acute exacerbations in children (78). However, this evidence is based primarily on cross-sectional studies of children who are clinically stable and from retrospective chart reviews. Evidence for the role of the five main bacteria in bronchiectasis is reviewed in the following sections, together with data on resistance to β-lactam and macrolide antibiotics, which are commonly used to treat respiratory infections.

**Table 1:** **Bacterial pathogens associated with bronchiectasis in children**.

Reference	Number	Setting	Age (years)	Specimen	*Haemophilus influenza* (%)	*Streptococcus pneumonia* (%)	*Moraxella catarrhalis* (%)	*Pseudomonas aeruginosa* (%)	*Staphylococcus aureus* (%)
Edwards et al. (2)	*n* = 60	New Zealand	1–17 (md 10)	Sputum	55 (NTHi)	10	5	2	0
Eastham et al. (71)	*n* = 93	United Kingdom	1.6–18.8 (md 7.2)	Various^a^	48	22	17	6	8
Karadag et al. (73)	*n* = 111	Turkey	1–17.5 (md 7.4)	Sputum	39	23	6	11	17
Li et al. (74)	*n* = 136	United Kingdom	3–18 (md 12.1)	Various^a^	39	17	2	11	4
Banjar (70, 120)	*n* = 151	Saudi Arabia	7.3 ± 4.1 (mean ± SD)	NP swab, sputum	37	17	9	16	7
Zaid et al. (75)	*n* = 92	Ireland	1.5–13 (md 6.4)	Sputum and BAL	54	37	10	9	15
Hare et al. (72)	*n* = 104	Australia (Indigenous)	0.4–12.9 (md 2.4)	BAL >10^4^ CFU/mL	31 (NTHi)	16	12	0	3
Kapur et al. (12)	*n* = 113	Australia	2.7–16 (md 5.3)	BAL ≥10^5^ CFU/mL	32 (NTHi)	14	8	2	5
Combined studies^b^	*n* = 860	Six countries	0.4–18.8	Various	40	20	8.5	7.9	7.6

*^a^ Cough swab, sputum, or BAL; ^b^ Studies differ in the specimens used for pathogen isolation and ways of reporting numbers or percentages therefore combined percentages are approximations only*.

*BAL, bronchoalveolar lavage; CFU, colony forming units; md, median; NP, nasopharyngeal; NTHi, non-typeable H. influenzae; sd, standard deviation*.

## Non-Typeable *Haemophilus influenzae*

*Haemophilus influenzae* is a Gram-negative pleomorphic coccobacillus and non-capsulated (non-typeable) strains (NTHi) are common colonizers of the upper respiratory tract (URT) (40). As the bacterium most commonly isolated from the airways of children and adults with bronchiectasis, NTHi likely contributes substantially to recurrent respiratory infections. In fact, the significance of NTHi in pediatric bronchiectasis was noted 60 years ago (79) with the conclusion that “non-capsulated *H. influenzae* is responsible for keeping the chronic inflammatory process smoldering in bronchiectatic individuals.” NTHi is also an opportunistic pathogen associated with other respiratory infections such as otitis media (OM), sinusitis, and pneumonia in children (80).

There are a number of virulence factors employed by NTHi that provide an environment conducive to infection and chronic colonization (41). Some of these, including production of biofilm and secretion of proteases, have been mentioned previously in this review. Biofilm has recently been described in children with bronchiectasis (67) and recent evidence from *in vitro* studies suggests that NTHi may manipulate activated neutrophils into facilitating the production of biofilm (81). NTHi also produces human IgA proteases that, *in vitro*, contribute to invasion of and survival within human respiratory epithelial cells (59).

Accurate identification of NTHi is important, since non-hemolytic strains of the closely related and primarily commensal, *Haemophilus haemolyticus*, may be misidentified as NTHi by phenotypic methods. Molecular detection methods found that 12–27% of nasopharyngeal (NP) isolates from healthy and otitis-prone children, initially identified as NTHi using phenotypic methods, were actually *H. haemolyticus* (82, 83). In Australian Indigenous children with bronchiectasis, most phenotypic NTHi isolates from the NP (87%) and BAL (88%) were confirmed as *H. influenzae* using *hpd*#3 PCR, whereas most oropharyngeal (OP) isolates (65%) were presumptive *H. haemolyticus* (84). NTHi lower airway infection was also confirmed by using the *hpd*#3 PCR quantitatively and comparing measures of bacterial density with total and differential cell counts to gage the airway inflammatory response to infection (85). These molecular studies differentiating NTHi from *H. haemolyticus* have reaffirmed the importance of NTHi as a lower airway pathogen in pediatric bronchiectasis and shown that the NP rather than the OP is the preferred site for NTHi carriage studies in this pediatric population.

Resistance to ampicillin and other β-lactam antibiotics in *H. influenzae* is generally limited to the production of β-lactamase or, in the case of β-lactamase negative ampicillin-resistant (BLNAR) strains, the presence of altered penicillin-binding proteins (PBPs) (86). The overall prevalence of β-lactamase positive respiratory tract strains was 16.6% in a large international surveillance study (PROTEKT) from 1999 to 2000, ranging from 3% in Germany to 65% in South Korea; only 0.07% of strains were BLNAR (87). In another large international study, most clinical *H. influenzae* strains were found to have an intrinsic macrolide efflux mechanism and azithromycin minimum inhibitory concentrations (MICs) between 0.25 and 4 mg/L; only 1.3% had high-level macrolide resistance (MIC >4 mg/L) due to ribosomal alterations, while 1.8% were defined as hyper-susceptible (MIC <0.25 mg/L) (88). In a cross-sectional study of 104 Australian Indigenous children with bronchiectasis, 19 and 33% of NTHi isolates from the NP and BAL, respectively, were β-lactamase positive and 6% and 13%, respectively, were azithromycin resistant (MIC >4 mg/L); all other isolates tested had intermediate azithromycin resistance (89).

### *Streptococcus* *pneumoniae*

*Streptococcus pneumoniae* (pneumococcus) is a Gram-positive diplococcus and the second most common pathogen associated with bronchiectasis in children (Table 1). At least 98 serotypes have been identified on the basis of its polysaccharide capsule (90). The capsule is an important virulence factor shielding the organism from the host’s immune system (91) and also an important vaccine target. Pneumococci colonize the nasopharynx of healthy individuals (92), but as opportunistic pathogens they can cause acute local infections, such as OM, sinusitis, and community-acquired bacterial pneumonia, or invade the bloodstream resulting in meningitis or sepsis. These acute pneumococcal infections and their complications have been studied extensively in children. Emerging evidence indicates a role for Th17 immune pathways in protection from mucosal infection with *S. pneumoniae* (93); however, little is known about the role of pneumococci in establishing and maintaining persistent lower airway or endobronchial infections in bronchiectasis. As severe and recurrent pneumonia episodes early in life are significant risk factors for bronchiectasis (37), it is plausible that early infection with *S. pneumoniae* initiates immunologic events that contribute to the pathogenesis of bronchiectasis.

From 1998 to 2000, the worldwide prevalence of pneumococcal pediatric respiratory tract isolates with high-level penicillin resistance (MIC ≥2 mg/L) was estimated at 18%, with conspicuous differences between countries (94). Pneumococcal resistance to β-lactams is primarily mediated through alterations in PBPs, with highly penicillin-resistant strains having more PBP alterations than strains exhibiting intermediate resistance (MIC 0.12–1 mg/L). Macrolide resistance in pneumococcal pediatric respiratory tract isolates worldwide was 25% overall (erythromycin MIC ≥1 mg/L), exceeding penicillin resistance in 19 of 26 countries surveyed (94). Macrolide resistance occurs through two main mechanisms; a ribosomal methylase and macrolide efflux system (95). Azithromycin is reported to be effective against susceptible strains of *S. pneumoniae*; however, the relevant MIC breakpoint is controversial and depends on the site of infection. Treatment failure has mostly been reported for community-acquired pneumonia (96), generally with MIC ≥8 mg/L (97). However, treatment failure has been reported for OM with MIC ≥2 mg/L (98). In the cross-sectional study of 104 Australian Indigenous children with bronchiectasis, 32 and 41% of *S. pneumoniae* isolates from the NP and BAL, respectively, were penicillin non-susceptible (no high-level penicillin resistance was detected) and 34 and 59%, respectively, were azithromycin resistant (azithromycin MIC ≥12 mg/L) (89).

In a randomized controlled trial (RCT) of weekly azithromycin versus placebo in 78 Indigenous children with bronchiectasis for up to 2 years, azithromycin resistance in *S. pneumoniae* from NP swabs was significantly higher in the azithromycin (79%) compared to placebo (8%) groups (99). However, resistance declined (to 7%) following the conclusion of the intervention (median 6 months later), indicating a fitness cost for macrolide resistance in *S. pneumoniae* (100). Similarly, studies of single-dose azithromycin and biannual mass treatments for trachoma have shown increased rates of pneumococcal macrolide resistance followed by a decline in resistance after removal of antibiotic pressure (101–103). This fitness cost of macrolide resistance in *S. pneumoniae* will likely ensure its eventual elimination (replacement of resistant strains by susceptible strains) in the absence of antibiotic selection (104).

### *Moraxella* *catarrhalis*

*Moraxella catarrhalis* is a Gram-negative, mostly β-lactamase positive, diplococcus. Although much of the research into pediatric respiratory conditions focusses on NTHi and pneumococcus, *M. catarrhalis* should not be overlooked. In a large cohort study of children presenting with cough, *M. catarrhalis* was the only pathogen that distinguished children with and without persistent cough 28 days later (105). *M. catarrhalis* is also a common cause of OM in infants and children and causes an estimated 2–4 million exacerbations of COPD in adults annually in the USA (106).

Most *M. catarrhalis* strains produce β-lactamase; 92% in the PROTEKT study (87). In contrast, *M. catarrhalis* is almost universally susceptible to azithromycin (87, 95, 107, 108), although macrolide resistance has recently been reported in China (109, 110), Japan (111, 112), and Pakistan (113). In Australian Indigenous children with bronchiectasis, most *M. catarrhalis* isolates from the NP (91%) and BAL (100%) were β-lactamase positive; however, azithromycin resistance was not tested (89). No data were located on macrolide resistance in *M. catarrhalis* from children with bronchiectasis. Given the increasing use of azithromycin for a range of conditions, it may be prudent to monitor *M. catarrhalis* for development of macrolide resistance.

### *Staphylococcus* *aureus*

*Staphylococcus aureus* is a Gram-positive coccal bacterium frequently found in the nose and on the skin and is a common cause of skin and respiratory infections. It is commonly associated with bronchiectasis in adults (76, 114). Although sometimes found in the BAL of children with bronchiectasis (Table 1), its role in pathogenesis is unknown.

Interest in *S. aureus* has intensified with the increasing prevalence of infections caused by methicillin-resistant *S. aureus* (MRSA) (115). MRSA is now endemic in many hospitals throughout the world, with the highest rates (>50%) in North and South America and south-east Asia (116). Studies of long-term macrolide treatment in cystic fibrosis patients with bronchiectasis have also found high rates of macrolide resistance (up to 100%) in *S. aureus* (117–119). We similarly found significantly higher macrolide resistance in *S. aureus* NP isolates from Indigenous children with bronchiectasis randomized to long-term azithromycin (100%) compared to placebo (40%); MRSA was present but less common in both treatment groups (19 and 20% of *S. aureus* isolates, respectively) (100). Importantly, all *S. aureus* isolates were macrolide resistant following the conclusion of the intervention (median 6-months later), providing the first evidence (to our knowledge) of a lack of fitness cost for macrolide resistance in this pathogen (100).

### *Pseudomonas* *aeruginosa*

*Pseudomonas aeruginosa* is a Gram-negative rod-shaped bacterium and opportunistic pathogen. *P. aeruginosa* is uncommon in young children with bronchiectasis (12, 72) but is found in older children (73, 74, 120) and adults with bronchiectasis (76, 114, 121, 122). Lower respiratory infection with *P. aeruginosa* is a predictor of accelerating lung function decline (28, 123), severe disease, and mortality (124), and is therefore likely associated with more advanced disease. Numerous mechanisms of antibiotic resistance have been attributed to *P. aeruginosa* including intrinsic and acquired β-lactamases and multidrug efflux pumps (125). No data were located on antibiotic resistance in *P. aeruginosa* from children with bronchiectasis.

## Initiation of Lower Respiratory Infection

Since lower airway infections result from aspiration of pathogenic bacteria originating in the upper airways (92), differences in URT carriage in different populations may be important. Four of the five main bacteria (*P. aeruginosa* being the exception) associated with bronchiectasis are common colonizers of the URT in children. Nasal or NP carriage of NTHi, *S. pneumoniae*, and *M. catarrhalis* is common in young children, particularly in day care centers (126, 127) and low-income countries (128). However, very high rates (80–90%) have been reported in Australian Aboriginal children (127, 129) and children in some developing countries such as Papua New Guinea (PNG) (130) and the Gambia (131, 132), which also have high rates of pneumonia morbidity and mortality (133, 134). Australian Aboriginal and Gambian infants acquire NP carriage of all three main respiratory pathogens at a very early age (132, 135), and the URT burden is higher in Indigenous than non-Indigenous Australian children (127, 136).

Nasopharyngeal carriage of *S. aureus* is high in infants <3 months old but declines rapidly as carriage of the three main respiratory bacteria increases, reaching a low point at 1–2 years (132, 137). Carriage increases again to reach its highest prevalence in children >5 years old (138, 139), as NP carriage of the other three bacteria declines. If aspiration of *S. aureus* from the URT contributes to bronchiectasis, then *S. aureus* lower airway infection may be more common in older children.

In contrast, *P. aeruginosa* is not considered part of the normal pharyngeal flora. In children with cystic fibrosis, carriage in the URT has only been found in those with established lower airway colonization (140). A recent study evaluated swabbing methods to estimate URT bacterial carriage and found very low prevalence of *P. aeruginosa* in all age groups (138). Aspiration of *P. aeruginosa* into the lower airways is thus likely a rare event, which may help to explain why this pathogen is especially uncommon in young children with bronchiectasis.

## Multiple Strain Carriage and Persistence of Infection

A notable feature of NTHi carriage in populations with high carriage rates is regular turnover of strains, and the multiplicity of NP strains carried at any one time (62, 130, 141). Populations with high *S. pneumoniae* carriage, such as Australian Aboriginal (141, 142), Gambian (131), and PNG children (130), also have high rates of simultaneous multiple serotype carriage; 10-fold higher than that reported in other populations (143). Pneumococcal serotypes are also regularly replaced; the mean duration of *S. pneumoniae* carriage in Kenyan children was just over 30 days (range 6.7–50 days for 28 serotypes) (144).

The high level of concordance between bacterial strains (NTHi ribotypes and pneumococcal serotypes) in the nasopharynx and lungs of Australian Indigenous children with bronchiectasis and concurrent carriage and lower airway infection (72) suggests recent aspiration of NP secretions, given the high turnover of NP strains. However, multiple strains of NTHi and *S. pneumoniae* have consistently been found more frequently in BAL compared to NP specimens (72, 89). This suggests accumulation of strains in the lower airways resulting from recurrent aspiration and failure to eliminate prior strains. Multiple NTHi strains have also been reported in sputum microbiology from adults with chronic respiratory conditions (54, 76).

**Figure 1:**
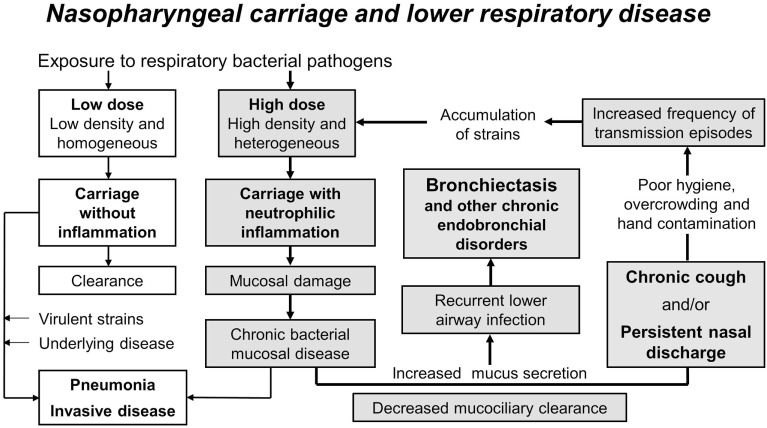
**The extended vicious circle hypothesis to explain high rates of chronic endobronchial disorders such as bronchiectasis**. The high and early burden of multiple pathogen species and strains in the nasopharynx of Indigenous children and children in low income countries (high-dose versus low-dose exposure) likely contributes to their high burden of acute and chronic lower respiratory infections. Carriage without inflammation is usually cleared but may result in pneumonia or invasive disease in the presence of virulent strains and/or underlying disease (unshaded boxes). Dense and diverse colonization causes neutrophilic inflammation, mucosal tissue damage, increased mucus secretion, and decreased mucociliary clearance, leading to chronic mucosal disease, recurrent lower airway infection, and bronchiectasis (shaded boxes). Persistent nasal discharge and chronic cough perpetuate this vicious circle, particularly where there is poor hygiene and overcrowding, by increasing opportunities for transmission. High rates of transmission events result in an accumulation of strains at a rate greater than can be cleared by the immune response (particularly in infants and young children) or by damaged mucosa, and the vicious circle (bold arrows) is repeated. Figure used with permission from *PNG Med J* (145).

It is probable that the high and early burden of pathogens in the nasopharynx of Indigenous children and children in low income countries contributes to the high burden of acute and chronic lower respiratory infections. Figure 1, adapted from Cole’s original model (10) and modified to explain chronic lung disease (145), illustrates an “extended vicious circle” hypothesis to explain high rates of chronic endobronchial disorders such as bronchiectasis.

## Challenges and Limitations

Despite what we have learned about the microbiology of the lower airways in children with bronchiectasis, there are challenges associated with identifying the pathogen responsible for a respiratory exacerbation. Spontaneous or induced sputum is a reliable and accessible source of specimen routinely used for microbiologic analysis in adults. However, collecting sputum from young children is problematic as they find it difficult to expectorate. Although NP swabs may be a useful proxy, in practice, the organism responsible for an exacerbation is rarely identified, and physicians generally rely on empiric evidence to treat respiratory exacerbations in children with bronchiectasis.

Contamination with upper airway microflora is a potential problem when lower airway specimens are obtained for bacterial culture (146). Despite careful technique, contamination may occur during BAL collection in young children as the tube used is too narrow for the protected brush method. Quantitative culture is used to exclude bacteria present in low numbers in BAL fluid due to upper airway contamination; however, the threshold used to define lower airway infection varies between studies. We and others have used a cutoff of 10^4^ colony forming units (CFU)/mL BAL (72, 147, 148) in children. However, others have used different cutoffs, e.g., 10^3^ CFU/mL in some adult studies (19, 146) and 10^5^ CFU/mL for infants with cystic fibrosis (149). Although we validated a threshold of 10^4^ CFU/mL to define NTHi lower airway infection using correlation with quantitative PCR and airway neutrophilia (85), alternative thresholds and other bacterial pathogens need investigation.

Identifying the pathogens responsible for lower airway infections is further complicated by limitations in laboratory methods. Only a small proportion of bacteria are detectable using routine laboratory culture and identification of respiratory pathogens from sputum or BAL alone does not prove causality of infection.

## Microbiome

Improvements in culture-independent molecular technologies show that the bronchial tree is host to a diverse microbiota, including respiratory pathogens, in people without respiratory illness (150). Thus, it is difficult to distinguish pathogenic colonization from commensal or opportunistic colonization in an individual patient. Nevertheless, members of the phylum Proteobacteria (which includes *Haemophilus* and *Moraxella* spp.) were strongly associated with airway disease (COPD and asthma) in adults and children (150).

The concept of dysbiosis (microbial imbalance) in intestinal flora has a long history and the associated literature is huge. In contrast, the healthy lung was long considered to be sterile, so that characterization of a healthy lung microbiome is a relatively recent development. Studies using 16S rRNA gene sequencing found that bacterial communities of the healthy lung overlapped those found in the mouth but at lower concentrations, while NP samples showed a different composition (151, 152). Bacteroidetes, particularly *Prevotella* spp., were frequently found in the mouth and lungs of healthy adults and children (150–152).

A recent study using 16S rRNA gene sequencing found that the microbiota in BAL specimens from 78 young children with chronic lung disease (36 had bronchiectasis) includes taxa present in both NP and OP specimens (153). Diversity using Simpson’s index was significantly lower in NP swabs compared to OP and BAL specimens, reflecting the more common dominance of individual operational taxonomic units (OTUs) in the NP microbiota (153). Dominant OTUs (>50% relative abundance) in NP and BAL specimens included *M. catarrhalis, H. influenzae, S. aureus*, and mitis group streptococci (which includes *S. pneumoniae*); only *Porphyromonas* sp. was dominant in OP specimens (153). These data support the hypothesis that the dynamics of bacterial populations in the nasopharynx are primarily responsible for dysbiosis in the lungs of children with bronchiectasis although both OP and NP analysis should be included in studies of the lower airways.

## Viruses

There are few data available with which to characterize the role of viral infection in the pathogenesis of bronchiectasis in children. However, recent data from two separate, prospective Australian studies of children with bronchiectasis indicate that attention to viruses is warranted. Respiratory viruses were associated with 48% of exacerbations in 69 Queensland children with bronchiectasis (154) and detected in the BAL of 44% of 68 clinically stable children, primarily Indigenous, in the Northern Territory (16). Furthermore, in a study of bronchiectasis in 58 adults in Guangdong, China, respiratory viruses were detected during 49% of 100 exacerbations (155). In the two pediatric studies, rhinovirus was the most commonly detected virus, while coronavirus, followed by rhinovirus and influenza were most common in the adult study. Whether this difference in viral dominance is demographically driven or associated with disease severity is unknown. However, in both children and adults, the presence of virus during respiratory exacerbation was associated with more severe symptoms.

Respiratory viruses are an important cause of exacerbations in other chronic respiratory illnesses including asthma and COPD (156, 157). It has been postulated that viruses may alter immune responses and promote respiratory exacerbations from bacterial infection (158). Furthermore, bacteria/virus coinfections reportedly result in more severe symptoms (158, 159). Australian Indigenous children carry a high burden of respiratory bacteria from a very young age (135, 160). Increased nasopharyngeal NTHi density has been shown in the presence of any one of several respiratory viruses in Indigenous children with acute OM; the most commonly detected viruses being rhinovirus, polyomavirus, and adenovirus (161). Adenovirus has also been associated with suppurative lung conditions in children, particularly with respect to bacterial coinfection (162).

Respiratory viruses are likely an under-recognized factor contributing to acute exacerbations and persistent airway inflammation in children with bronchiectasis. Large, population-based pediatric studies investigating the effect of viruses on airway immunopathology are important to fully appreciate the contribution of viruses to chronic inflammation and the pathogenesis of bronchiectasis in children.

## Impact of Antibiotics

Antibiotic therapy forms the cornerstone of bronchiectasis management in children. Antibiotics are prescribed to reduce symptoms, prevent exacerbations, and preserve lung function by reducing lower airway bacterial load and inflammation (163). Amoxicillin remains the antibiotic of choice for acute respiratory infections due to its long history of clinical success, acceptability, limited side effects, and relatively low cost. High doses of amoxicillin can be prescribed for penicillin-resistant pneumococci, but clinical failure may indicate another infecting pathogen that is a β-lactamase producer. This mode of resistance can be overcome by antibiotics containing β-lactamase inhibitors such as clavulanic acid. Oral amoxicillin-clavulanate is usually prescribed as initial empiric therapy for children with bronchiectasis and mild-to-moderate exacerbations (163).

The macrolide antibiotic azithromycin is often used long term for the management of bronchiectasis in children and adults (8). Azithromycin does not require refrigeration and can be taken orally once a week and is therefore more easily managed in resource poor settings. Azithromycin has been used in pediatric patients since 1991 and has been found to be safe and well-tolerated in the single-dose regimen as well as the conventional 3-day and 5-day regimens (164). Macrolides are active against *S. pneumoniae, H. influenzae, M. catarrhalis*, and *S. aureus* (165, 166); they have no bactericidal effects against *P. aeruginosa* but do inhibit biofilm formation (166). Macrolides have immunomodulatory properties and antibacterial effects (166), decreasing inflammation, inhibiting bronchial hyper-responsiveness, and improving mucus clearance (167), and may therefore play an important role in the management of bronchiectasis.

Three reviews (166, 168, 169) of maintenance macrolide therapy (2–24 months duration) for bronchiectasis have reported six RCTs in adults and four in children (99, 170–172). Macrolide therapy was associated with reduced frequency of exacerbations in adults (risk ratio [RR] 0.42, *P* < 0.001) and children (RR 0.50, *P* < 0.001) (169), often with other improvements in pulmonary function and reduced sputum volume. Only one RCT of low-dose erythromycin versus placebo in children with HIV-related bronchiectasis found no difference in the frequency of exacerbations (172). Increased macrolide resistance in NP bacteria (*S. pneumoniae* and *S. aureus*) was found in the only RCT in children which detailed microbiologic outcomes (99). However, adherence to medication in the Australian azithromycin group was significantly associated with lower carriage of any pathogen and fewer macrolide-resistant pathogens (95% of New Zealand children took ≥70% of their weekly doses as directly observed therapy was practiced) (100). Better adherence to treatment will not only optimize clinical benefit (99) but also by lowering the bacterial load and minimizing prolonged periods of sub-MIC antibiotic concentrations, it may also reduce the risk of macrolide resistance (100). This has important implications for physicians when considering long-term macrolide antibiotics for children with bronchiectasis, since adherence <70% was a significant risk factor for macrolide resistance (100).

## Impact of Vaccines

Currently, there is no commercially available vaccine specifically targeting NTHi, the main bacterial pathogen in pediatric bronchiectasis. Oral vaccines against infection with NTHi have been successful in reducing the number and severity of exacerbations, as well as carriage, in adults with COPD and chronic bronchitis (173–176). However, a systematic review found the benefit was too small to advocate widespread NTHi oral vaccination of people with COPD (177). A more effective oral vaccine may improve mucosal protection (178) and reduce the incidence and/or severity of respiratory infections caused by NTHi.

Pneumococcal polysaccharide vaccines were developed to prevent pneumonia caused by *S. pneumoniae* and include serotypes causing the most invasive pneumococcal disease (IPD). The 23-valent polysaccharide vaccine was introduced in 1983 (179) and is still in use today. However, while the antibody response to immunization with polysaccharide is satisfactory in most people over the age of two, the response is less satisfactory between 6 months and 2 years of age and very poor in children < 6months old (180). Conjugation of pneumococcal polysaccharides to a carrier protein improves immune responses among infants. The first licensed pneumococcal conjugate vaccine (PCV7) introduced in 2000 contained seven serotypes causing most IPD in children <5 years old in the USA (181). Vaccines including more serotypes have subsequently been produced, including a 10-valent pneumococcal NTHi protein D conjugate vaccine (PHiDCV) (182) and PCV13, licensed in 2010 (183).

PCVs have been very effective in reducing the incidence of IPD caused by vaccine serotypes (184–186). However, PCVs have been less effective in reducing pneumonia (a risk factor for bronchiectasis); reductions in radiologically confirmed (likely bacterial) pneumonia of 20–22% have been described in some populations (187, 188) but no reduction in others (189). Limited impact on pneumonia incidence may be due to the relative contribution of vaccine-type pneumococci in a setting where multiple other pathogens (including pneumococcal serotypes not in the vaccine) are prevalent.

There are few published clinical trials assessing the impact of PCVs in children with bronchiectasis, although vaccination is currently recommended (78). A clinical trial of PHiDCV in children with protracted bacterial bronchitis, chronic suppurative lung disease, and bronchiectasis (collectively referred to as chronic endobronchial disorders), with respiratory exacerbation as the primary outcome, is currently underway (190). We have recently found that carriage serotypes of *S. pneumoniae* (mostly non-vaccine types in PCV-vaccinated populations) have a similar propensity to cause lower airway infection in Australian children with chronic endobronchial disorders (191). From these data, it may be argued that PCVs will have little impact on lower respiratory infection in this population. However, PHiDCV may be effective against mucosal infection with NTHi (192), and we await the full findings of the clinical trial (190).

Further molecular studies are required, since not all *H. influenzae* strains have the *hpd* genes encoding *Haemophilus* protein D (193). This has implications for *H. influenzae* identification, and other gene targets have been used (194, 195). There are also important implications for vaccine targeting, and studies are needed to determine the prevalence of *hpd* negative strains in different populations and different sites of carriage and infection and to investigate other potential NTHi vaccine antigens.

Vaccines targeting the other three main bacterial pathogens in pediatric bronchiectasis, *M. catarrhalis, P. aeruginosa*, and *S. aureus* are not yet commercially available; their development has been reviewed by others (196–198).

## Anti-Inflammatories

Inhaled anti-inflammatories may be a strategy to manage the intense and persistent airway inflammation associated with bronchiectasis. Inhaled non-steroidal anti-inflammatories (NSAIDs) and corticosteroids have been proposed as potential agents in the management of bronchiectasis (8). However, two recent systematic reviews of randomized, controlled clinical trials found no evidence to support the use of inhaled NSAIDs (199) or corticosteroids (200) in children with bronchiectasis. These reviews emphasize a gap in knowledge regarding novel therapeutics to address airway inflammation in children with bronchiectasis. There is a need for clear data regarding the effect of inhaled anti-inflammatories on airway inflammation and the long-term effect of anti-inflammatories on recurrent infection in children with bronchiectasis, prior to introducing them into the therapeutic regime.

## The Road Forward

The past decade has seen tremendous advances in our understanding of the immunologic parameters associated with bronchiectasis. Bronchiectasis is a complex condition involving suboptimal adaptive immune responses and dysregulated inflammatory responses that culminate in recurrent and persistent infection. However, current management strategies for children continue to rely primarily on antibiotic therapy for the treatment or prevention of acute bacterial infection and physiotherapy for airway clearance. While these strategies are important, a novel, multidirectional approach is required to address the impaired adaptive immune responses and dysregulated inflammatory mechanisms responsible for chronic inflammation and persistent infection. Novel, plausible therapeutic directives may include targeting airway inflammation through the use of inhaled anti-inflammatories or improving adaptive immune responses to pathogens important to the pathogenesis of bronchiectasis. However, both approaches require a commitment to understanding the complex immunology associated with suboptimal immune responses and protective immune pathways, prior to developing and incorporating immunomodulating therapeutics to the management of bronchiectasis in children. Understanding the mechanisms driving the immunopathology of bronchiectasis has the potential to revolutionize therapeutics and management strategies for children. Here, we consider future research endeavors to advance management and prevention of bronchiectasis in children.

### Developing Non-Invasive Methods to Define and Monitor Airway Inflammation

Rapid advancements in therapeutics against persistent inflammation are hindered by difficulties in monitoring their effect on airway inflammation. Obtaining lower airway specimens from children is challenging. Young children find it difficult to expectorate until at least 7 years of age and bronchoscopy with lavage is an invasive procedure performed under anesthesia. Non-invasive approaches for monitoring inflammation in the lungs, including the resolution of inflammation, are required. Approaches that warrant investigation include the use of exhaled breath condensate (201) and biomarkers from upper airway mucosal secretions (202) and blood (23) reflective of lower airway inflammatory processes.

### Understanding the Mechanisms Responsible for Immune Dysfunction

Dysregulated airway inflammation appears to be linked to the functional phenotype of the adaptive immune response (16); however, the direction of this association, be it cause or effect, is unknown and likely complex and questions remain regarding the transition between acute and chronic responses. Inappropriate T-cell responses may increase susceptibility to infection with NTHi and prolong inflammation due to ineffective clearance mechanisms. Dysregulated IL-6 and IL-1β pathways in the lungs may promote an environment supportive of Th2 and Th17 polarized responses, increasing susceptibility to infection with NTHi and viruses. It is also plausible that chronic inflammation may contribute to impaired macrophage activation and T-cell responses, resulting in an environment of immune tolerance or exhaustion. Understanding immunologic mechanisms behind the chronic symptoms of bronchiectasis will greatly inform targeted management practices.

### Improving Immunity to NTHi

Limited data suggest that it may be possible to improve immunity to NTHi in children with bronchiectasis. Vaccination with a PCV containing a single-NTHi antigen was associated with higher NTHi-driven IFN-γ responses (51). While it appears that a strong IFN-γ response is important for immunity to NTHi, the specific cytokine profile that correlates with protection is unknown. Indeed, while the IFN-γ levels produced by children with bronchiectasis who received the vaccine approached levels obtained by healthy control children, the children with bronchiectasis also produced higher levels of Th2-associated cytokines (IL-13 and IL-5), suggesting generation of a mixed Th1/Th2 response. As mixed Th1/Th2 responses are associated with suboptimal protection from pertussis (203), effective vaccine development will rely on understanding the functional cell-mediated phenotype that best correlates with protection from infection with NTHi.

### Does Immune Dysfunction Extend to Pathogens Other Than NTHi?

Non-typeable *Haemophilus influenzae* is the most common known pathogen identified in the lower airways of children with bronchiectasis; however, other respiratory pathogens, including viruses, are also common. Furthermore, accumulating data indicate that the airways are host to a diverse microbiota, including respiratory pathogens, in people without respiratory disease. Thus, distinguishing infection from opportunistic colonization can be difficult. It has been proposed in the literature that respiratory disease may be better characterized by changes in the phenotypic profile of the microbiota (150). Detailed data investigating the relative effect of changes in the lung microbiota on immune function in children are lacking. Such studies would provide important information regarding immune development and the pathogenesis of bronchiectasis.

### Bacterial Load Thresholds to Define Lower Airway Infection

Respiratory societies have introduced guidelines for collecting BAL fluid for microbiologic and immunologic analysis. However, instrumentation, technique, and sample preparation can vary widely between centers and between individual operators. The effect this has on the phenotype or quality of the sample must be considered when reporting results. Although we have validated a threshold of 10^4^ CFU/mL to define NTHi lower airway infection in our setting (85), alternative thresholds should be investigated. Multiple biologic and clinical markers consistent with disrupted airway homeostasis should be considered when developing algorithms to define thresholds of infection. Further, the association between bacterial load and inflammatory markers may differ between bacterial species. Well-defined thresholds of infection that can account for operational variables are important for understanding the contribution of each pathogen to lower airway inflammation in children with bronchiectasis.

### Genome Studies

Whole genome sequencing (WGS) is needed to understand virulence and antibiotic resistance determinants in important bacterial pathogens. We have reported higher rates of resistance in BAL compared to NP isolates for NTHi, *S. pneumonia*, and *M. catarrhalis* (89). Lower airway strains may harbor a significantly higher proportion of genetic variants that favor persistence or confer antimicrobial resistance compared to NP strains from the same children and/or asymptomatic carriers. Further, WGS studies are needed to determine the prevalence of *hpd*-negative NTHi strains in different populations and different sites of carriage and infection and to investigate other potential vaccine antigens in NTHi and other pathogens. This information may prove valuable in the development of vaccines and therapeutic agents.

## Concluding Statement

Suboptimal adaptive immune responses, in addition to dysregulated local inflammatory responses, likely contribute to an environment conducive to chronic or recurrent infection. An effective management strategy for bronchiectasis in children requires an understanding of the adverse immunologic events leading to recurrent infection and persistent inflammation. Promoting an environment that supports efficient pathogen clearance and rapid resolution of inflammatory responses should be forefront in our future endeavors to combat a condition that should be largely preventable.

## Author Contributions

SP, KH, and JU wrote and edited the review.

## Conflict of Interest Statement

The authors declare that the research was conducted in the absence of any commercial or financial relationships that could be construed as a potential conflict of interest.
